# Biochemical role of FOXM1-dependent histone linker H1B in human epidermal stem cells

**DOI:** 10.1038/s41419-024-06905-1

**Published:** 2024-07-17

**Authors:** Maria Pia Polito, Grazia Marini, Alessandra Fabrizi, Laura Sercia, Elena Enzo, Michele De Luca

**Affiliations:** Centre for Regenerative Medicine “Stefano Ferrari”, Department of Life Science, University of Modena and Reggio Emilia, Modena, Italy

**Keywords:** Skin stem cells, Transcription factors

## Abstract

Epidermal stem cells orchestrate epidermal renewal and timely wound repair through a tight regulation of self-renewal, proliferation, and differentiation. In culture, human epidermal stem cells generate a clonal type referred to as holoclone, which give rise to transient amplifying progenitors (meroclone and paraclone-forming cells) eventually generating terminally differentiated cells. Leveraging single-cell transcriptomic data, we explored the FOXM1-dependent biochemical signals controlling self-renewal and differentiation in epidermal stem cells aimed at improving regenerative medicine applications. We report that the expression of H1 linker histone subtypes decrease during serial cultivation. At clonal level we observed that H1B is the most expressed isoform, particularly in epidermal stem cells, as compared to transient amplifying progenitors. Indeed, its expression decreases in primary epithelial culture where stem cells are exhausted due to FOXM1 downregulation. Conversely, H1B expression increases when the stem cells compartment is sustained by enforced FOXM1 expression, both in primary epithelial cultures derived from healthy donors and JEB patient. Moreover, we demonstrated that FOXM1 binds the promotorial region of H1B, hence regulates its expression. We also show that H1B is bound to the promotorial region of differentiation-related genes and negatively regulates their expression in epidermal stem cells. We propose a novel mechanism wherein the H1B acts downstream of FOXM1, contributing to the fine interplay between self-renewal and differentiation in human epidermal stem cells. These findings further define the networks that sustain self-renewal along the previously identified YAP-FOXM1 axis.

## Introduction

Histones constitute the primary components of mammalian nuclei, organizing into nucleosomes. While histones H2A, H2B, H3, and H4 form the core of the nucleosome, the H1 linker histones envelop the DNA around the core, creating the characteristic “beads on a string” structure [1]. Recent insights have underscored their broad impact beyond mere chromatin compaction, revealing specific functions in gene regulation, DNA repair, and cell cycle progression [2].

In mammals, 7 somatic H1 subtypes have been identified—H1A (H1.1), H1B (H1.5), H1C (H1.2), H1D (H1.3), H1E (H1.4), H10, and HIX—encoded by the *HIST1H1A*, *HIST1H1B*, *HIST1H1C*, *HIST1H1D*, *HIST1H1E*, *H1F0* and *HIFX* genes, respectively [3].

H1 linker histones feature a tripartite domain structure: a central globular domain (GD) characterized by a winged-helix motif, alongside less conserved N-terminal tail domain (NTD) and lysine-rich C-terminal tail domain (CTD) [4]. Notably, while H10 and H1X share only 45% and 37% sequence identity with other somatic H1 histones, H1B, H1C, H1C, and H1E exhibit higher homology (ranging between 76% and 86%), suggesting potential functional relatedness [2]. Emerging evidence suggests divergent biochemical properties among these subtypes, including variations in chromatin affinity, compaction effects, and partner binding. For instance, H1B, H1D, and H1E demonstrate higher chromatin affinity, as compared to H1A and H1C [5, 6], even if post-translational modifications and histone chaperones affect their biochemical properties [7, 8].

Experimental studies, particularly in mice, have revealed compensatory mechanisms and functional redundancy among different H1 subtypes, evident during embryogenesis and in adult tissues [2]. H1B has been implicated in myogenesis inhibition through repression of the MyoD gene promoter in C2C12 myoblasts [9]. Instead, H1C and H1E are mainly related to progression of differentiation in retinal cells, thymocyte and intestine [10, 11]

Human keratinocyte primary cultures have been used for decades to permanently regenerate a functional epidermis or a corneal epithelium in burns victims [12–14]. More recently, transgenic epidermal cultures have been successfully used as combined cell and gene therapy to restore the epidermis in patients suffering from severe forms of junctional epidermolysis bullosa (JEB) [15–18]. Hence, primary epidermal cultures stand as an ideal model for investigating H1 linker dynamics during human epidermal self-renewal and differentiation.

Human clonogenic keratinocytes give rise to three clonal types, referred to as holoclones, meroclones and paraclones [19, 20]. Holoclones are generated by long-lived, self-renewing epithelial stem cells while meroclones and paraclones are originated by transient amplifying (TA) progenitors [16]. Transcriptomic profile of the three clonal types has been previously defined by combination of microarray and single-cell sequencing [21]. This characterization led to the identification of genes involved in self-renewal regulation of human epidermal stem cells. Cell cycle, DNA repair, microtubule organization, and YAP and FOXM1 pathways are differentially expressed between epidermal stem cells and TA progenitors [21–25].

Epigenetic regulation of genes associated with self-renewal and differentiation is emerging as a crucial mechanism in epidermal stem cells. P63, a key transcription factor regulating both epidermal development and proliferative potential of squamous epithelial stem cells [14, 26–29], interacts with chromatin remodelers to increase chromatin accessibility and activates the lncRNA XP33, which is involved in the expression of the late cornified gene LCE2D [30, 31]. Furthermore, DNA methylation, histone modification, and chromatin remodelling play significant roles in epidermal differentiation [32]. DNMT1, expressed in the basal layer of the epidermis, is necessary for DNA methylation of non-epidermal genes during stem cell differentiation [33]. Functional studies in mouse models have also identified that Ezh1 and Ezh2 prevent the binding of AP1 to genes encoding proteins involved in epithelial barrier formation [34, 35]. Components of the SWI/SNF chromatin remodelling complex, such as BRG1, BRM, BAF, and ACTL6A, are essential for establishing the correct epigenetic state of the epidermal differentiation complex (EDC) genes [36–38].

In this study, we focused on histone linker H1B as one of the most differentially expressed genes between epidermal stem cells and TA progenitors and delve into its role in the epigenetic control of the balance between self-renewal and differentiation in epidermal cells.

## Methods

### Encapsulation with 10X Genomics chromium system, single-cell RT and bioinformatic analysis in single-cell RNA seq data

For details on single-cell RNA sequencing experiments refer to [21]. Briefly, two keratinocyte cultures were detached with trypsin for 15–20 min in order to obtain a single cell suspension, pelleted and resuspended in culture medium and in 1× phosphate-buffered saline (PBS) with 0.04% BSA. 10,000 cells of each sample were loaded into one channel of the Chromium Chip B using the Single Cell reagent kit v3 (10X Genomic). cDNA was synthesized and amplified for 14 cycles following the manufacturer’s protocol. Sequencing was performed on the NextSeq550 Illumina sequencing platform, reaching at least 50,000 reads as mean reads per cell. The Cell Ranger Count pipeline (version 3.1.0) was used to align reads of the dataset to the reference transcriptome (GRCh38) and to calculate UMI counts from the mapped reads. Expression data were imported in R and analysed using Seurat (version 4.3.0) R package. 3.367 and 3.978 cells were obtained from two independent sub-confluent primary epidermal cultures.

Single-cell transcriptomic data of healthy-donor-derived skin biopsy were processed using the same procedure described above, retrieving 8570 cells. Cells were classified using the annotated dataset as reference and the *FindTransferAnchors* and *TransferData* functions in Seurat with default parameters. Seurat *MapQuery* function was used to project the healthy-donor-derived skin biopsy dataset onto the reference UMAP structure.

For the co-expression analysis, data were normalized using the *NormalizeData* function in Seurat. Expression levels for FOXM1 and HIST1H1B were extracted, with an expression threshold set at >0. Colocalization was identified when the expression levels of both genes exceeded this threshold. Starting from the normalized data, the average expression for each cluster was calculated using the *AverageExpression* function in Seurat.

### Human tissues

All human tissues were collected after informed consent for use of tissues in research and in compliance with Italian regulations (Comitato Etico dell’Area Vasta Emilia Nord, number 178/09 for healthy donor skin samples and number 124/2016 skin biopsies obtained from patients affected by JEB).

### Primary human cell cultures from healthy donors and JEB patients

Human skin samples from surgical waste (abdominoplasty or mammoplasty) were collected and anonymized. Briefly, skin biopsies were minced and treated with 0.05% trypsin/0.01% EDTA for 4 h at 37 °C. Every 30 min keratinocytes were collected, plated (2.5–3 × 10^4^/cm^2^) on lethally irradiated 3T3-J2 cells (2.4 × 10^4^/cm^2^), and grown at 37 °C, 5% CO_2_ in humidified atmosphere in Dulbecco’s modified Eagle’s (DMEM) and Ham’s F12 media (2:1 mixture) containing fetal bovine serum (FBS) (10%), penicillin–streptomycin (50 lU/ml), glutamine (4 mM), adenine (0.18 mM), insulin (5 mg/ml), cholera toxin (0.1 nM), hydrocortisone (0.4 mg/ml), triiodothyronine (Liothyronine Sodium) (2 nM), epidermal growth factor (EGF, 10 ng/ml) (Kc). When sub-confluent, cell cultures were serially propagated until senescence. For further details, see ref. [20].

A skin biopsy (1 cm^2^) has been collected from a LAMB3-dependent JEB patient (1-month-old) and cultivated as described above.

### 3T3-J2 cell line

Mouse 3T3-J2 cells were a gift from Prof. Howard Green, Harvard Medical School (Boston, MA, USA). Fibroblasts were cultivated in DMEM supplemented with 10% gamma-irradiated donor adult bovine serum, penicillin–streptomycin (50 IU/ml) and glutamine (4 mM). EUFETS, GmbH (Idar-Oberstein, Germany) produced a GMP clinical grade 3T3-J2 cell bank [39]. That have been authorized for clinical use by national and European regulatory authorities.

### Clonal analysis

Sub-confluent keratinocytes mass cultures were trypsinized and 0.5–1 cell was plated into each well of a 96-well plate after serial dilution. Single clones were cultivated for 7 days and treated with 0.05% trypsin and 0.01% EDTA at 37 °C for 15–20 min. One-quarter of the clone was plated into an indicator dish, cultivated for 12 days, and stained with rhodamine B to classify the clonal type. The remaining three-quarters were sub-cultivated into an adequate plastic support and used for further analyses [20].

### Plasmid constructs

For FOXM1 overexpression, cDNA of FOXM1-C isoform was cloned in pCDH1 expression plasmid under the control of a constitutive CMV promoter (gift from Weiguo Hu). Empty backbone was used as control. Inducible pTRIPZ lentiviral shRNA vectors were purchased from Dharmacon.

### Lentiviral production and primary human keratinocyte infection

HEK293T cells were cultivated in DMEM supplemented with 10% FBS, 1% Pen/Strep, 1% glutamine (Thermo Fisher). HEK293T cells were transiently transfected with pMD2-VSVG, pPAX2, and the lentiviral plasmid by using calcium phosphate transfection. Lentiviral particles were collected after 48 h post-transfection, filtered through 0.45-μm-pore cellulose acetate filters, and concentrated by ultracentrifugation. Primary human keratinocytes were transduced with 8 mg/ml polybrene (Sigma) at MOI 10. Keratinocytes were passaged at subconfluence and collected for further analyses.

### Western blotting

Feeder layer was removed in 20 mM cold PBS/EDTA. Keratinocytes were collected by scraping in 1× RIPA buffer (Sigma Aldrich) supplemented with Phosphatase and Protease Inhibitor Cocktails (Thermo Fisher). Pierce BCA Protein Assay kits (Thermo Scientific) were used to quantify the total protein amount. The same amount of proteins was loaded in 4–12% NuPAGE Bis-Tris Gels and transferred 100 V at 4 °C for 2 h onto nitrocellulose membrane (Millipore). Membranes were treated with Everyblot blocking solution (Bio-Rad). Primary antibodies were diluted in Everyblot blocking solution (Bio-Rad) as indicated in Supplementary Table 1 and added overnight at 4 °C to the membranes. Secondary antibodies were diluted in Everyblot blocking solution (Bio-Rad) as indicated in Supplementary Table 1 and added to the corresponding membranes for 1 h at room temperature. Signal was visualized with Clarity Western ECL substrate (Bio-Rad) using ChemiDoc (Bio-Rad) and ImageLabs software. Grey background on the images was homogeneously added for graphical purpose.

### Transient transfection

A total amount of 100 nM of specific siRNA (Silencer Select, Thermo Fisher; Supplementary Table 2) were transfected by Lipofectamine RNAiMAX (Thermo Fisher) for 5 h in the absence of serum. After 5 h the medium was changed and replaced in Kc medium. The cells were collected at the indicated time after transfection in RIPA buffer (Sigma) for protein extract or in Lysis buffer (Thermo Fisher) for mRNA collection.

### Binding sites analysis

To predict potential binding sites for the transcription factor FOXM1 in the promoter region of the *HIST1H1B* gene, the Biostrings package on R was used. FOXM1 binding motif was retrieved from Jaspar website (https://jaspar.uio.no/) (ID: UN0802.1). Promoter sequence of the *HIST1H1B* gene was obtained from UCSC Genome Browser (1 kb upstream TSS). *MatchPattern* function was used to scan for FOXM1 motif occurrences in the promoter sequence, allowing a maximum mismatch = 1.

### Chromatin immunoprecipitation

For chromatin immunoprecipitation iDeal ChIP-seq kit for Transcription Factors from Diagenode (Cat. No.: C01010055) was used following the manufacturer’s instruction. Briefly, cells were crosslinked with 1% formaldehyde (Sigma) in culture medium for 10 min at room temperature, and chromatin from lysed nuclei was sheared to 200–600 bp fragments using a Branson Sonifier. Chromatin derived from 4 × 10^6^ cells was incubated with indicated antibodies (Supplementary Table 1) overnight at 4 °C. Antibody/antigen complexes were recovered with ProteinA/G beads for 2 h at 4 °C. Quantitative real-time PCR was carried out on a QuantStudio 12k Flex Real-Time PCR System (Thermo Fisher) with custom-made oligonucleotides and PowerUP SYBR green master mix (Thermo Fisher); each sample was analysed in triplicate. The amount of immunoprecipitated DNA in each sample was determined as the fraction of the input (amplification efficiency (Ct INPUT_Ct ChIP)). Primers are listed in Supplementary Table 3.

### RNA extraction and real-time qPCR

For real-time qPCR, total RNA was isolated from cultured cells using the PureLink RNA Mini Kit (Thermo Fisher). Complementary DNA was generated using the SuperScript VILO cDNA Synthesis Kit (Thermo Fisher). Real-time qPCR analyses were carried out on triplicate samplings of retrotranscribed cDNAs with Taqman Universal PCR Master mix or PowerUP SYBR green master mix (Thermo Fisher) on QuantStudio 12k Flex Real-Time PCR System (Thermo Fisher). Expression levels are given relative to GAPDH. List of TaqMan probes (Thermo Fisher) and oligonucleotide custom made by Eurofins Genomics is provided in Supplementary Tables 3 and 4, respectively. Data were analysed with RQ Manager Software 1.2.2 and visualized with Prism 8.

## Results

### H1B is mainly expressed in epidermal stem cells

**Fig. 1:**
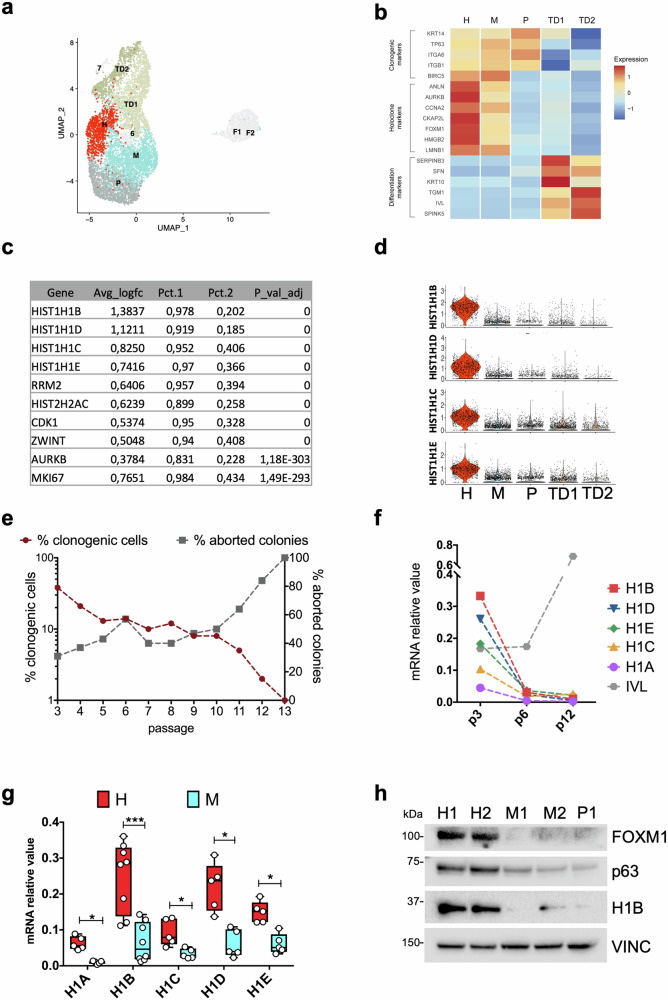
Single-cell transcriptomic data reveal that H1B is mainly expressed in epidermal stem cells. **a** Uniform manifold approximation and projection (UMAP) of the single cell RNA-seq dataset of 3.367 and 3.978 cells obtained from two sub-confluent primary epidermal cultures derived from independent healthy donors derived from Enzo et al. [21]. Keratinocyte clusters (H, M, P, TD1, TD2) are coloured according to cluster identity. Fibroblasts and low-quality clusters (F1 and F2, 6, 7) are shown in light grey. **b** Heatmap showing expression of clonogenic, holoclone, and differentiation markers used to annotate the five keratinocyte clusters. **c** Table showing the first ten most differentially expressed genes in H vs M clusters. **d** Violin plots showing the expression of *HIST1H1B, HIST1H1D, HIST1H1C* and *HIST1H1E* among the 5 clusters identified with scRNA-seq. **e** Serial cultivation of normal human keratinocytes (NHK). Percentage of clonogenic cells (dark red line) was calculated as the ratio between grown colonies and plated cells. Percentage of aborted colonies (grey line) was calculated as the ratio between the colonies scored as aborted and the number of clonogenic cells. **f** qRT-PCR quantification of the mRNA levels of five different H1 isoforms (*H1A, H1B, H1C, H1D, H1E*) and one differentiation marker (*IVL*) on NHK collected during serial cultivation. Expression levels were normalized per *GAPDH*. Data from one representative experiment are shown. **g** qRT-PCR quantification of the mRNA levels of five different H1 isoforms (*H1A, H1B, H1C, H1D, H1E*) on clones generate from at least two primary cultures. Expression levels were normalized per *GAPDH*. Holoclones and Meroclones are displayed in red and light blue, respectively. Data are presented as mean +/− SD, *N* = 5 different independent biological replicates, *P < 0.05, ***P < 0.001 Student t-test. **h** Western analysis of total cell extracts from cultures generated by holoclones (H1 and H2), meroclones (M1 and M2), and paraclone (P1) isolated by clonal analysis (see ‘Methods’) of sub-confluent NHK. Molecular weight indicators are shown.

Primary human keratinocytes were isolated from biopsies taken from healthy donors and cultivated onto lethally irradiated murine fibroblasts, as described under ‘Methods’ [20]. This procedure ensures the proper expansion of epidermal stem cells—eventually leading to the generation of TA progenitors and differentiated cells—and the clonal analysis of the different clonal types [16, 20, 21]. To gain deeper insights into the biochemical signals driving self-renewal and differentiation in these cells, we leveraged our previously published transcriptomic dataset. The single-cell transcriptomic profile of cultured human epidermal cells identified 5 clusters. Cell cycle regression has been applied during the analysis, in order to avoid any bias linked to it. Three clusters (designated as H, M and P) expressed markers characteristics of clonogenic cells, such as *ITGB4, ITGB1, KRT14, TP63, BIRC5*, while the other two clusters (TD1 and TD2) expressed genes associated to the epidermal differentiation, such as *14-3-3-σ, KRT10, SPINK5, TGM1, IVL* (Fig. 1a, b, and ref. [21]). The H cluster exhibited the highest expression of the “holoclone signature” previously identified as the hallmark of holoclone-forming stem cells, whilst M and P clusters identify TA progenitors. The holoclone signature progressively decreases during H to M/P clonal conversion and is almost absent in the differentiated TD1/2 clusters [21].

In this study, our aim was to identify genes closely related to self-renewal potential, specifically marking cells in the H cluster. We considered only those genes expressed in more than 80% of the cells derived from the H cluster and in less than 30% of the cells in the M cluster. Among the top genes differentially expressed between the H and M clusters, we identified the H1 linker histone genes *H1B*, *H1D*, *H1C*, and *H1E* (Fig. 1c). Violin plots depicting the expression of these genes in each cluster showed their predominant expression in the H cluster, with minimal expression observed in other clusters (Fig. 1d). Regarding alternative H1 linker isotypes, while *H10* was not expressed in human primary keratinocytes, *H1F* did not exhibit differential expression, and *H1A* showed only partial differential expression (Supplementary Fig. 1a).

To address potential biases associated with the capture of poly-A lacking transcripts in the 10X Genomics protocol, we assessed the mRNA levels of these transcripts by real-time PCR where retrotranscription is driven by random primers (see ‘Methods’). First, we analysed the expression of Histone H1 genes in human primary keratinocytes during serial cultivation: in early passages, the culture is enriched in stem cells that, during serial passaging, progressively give rise to TA progenitors and, eventually, to terminally differentiated cells; consequently, by passage 12 the culture is mainly composed of terminal colonies (Fig. 1e and Supplementary Fig. 1b, c). Histone H1 subtypes mRNA levels decreased during cell passages (p3, p6 and p12), while a differentiation marker as *IVL*, increased during serial cultivation (Fig. 1f and Supplementary Fig. 1d, e). To confirm that histone linkers are predominantly expressed in the stem cell compartment, keratinocyte cultures from at least two healthy donors underwent clonal analysis. This method allowed the isolation of a single clones derived from a single cell plated seven days earlier. The analysis of cell progeny obtained after single clone propagation, enabled the classification of the original cell as holoclone-forming cell (i.e. stem cell) or meroclone and paraclone-forming cells (i.e. TA progenitors) [20]. Real-time analysis on RNA extracted from the clone progeny confirmed the expression levels of H1 linker subtypes. *H1B* and *H1D* mRNA exhibited the highest expression in holoclones, as compared to the lower expression of *H1E, H1C* and *H1A* (Red bars, Fig. 1g). Clonal conversion, reflecting the transition from holoclones to mero/paraclones, corresponded to a substantial decrease in H1 linker mRNA levels (light blue bars, Fig. 1g). Western blot analysis confirmed the high expression of H1B in holoclones and its minimal expression in meroclones and paraclones (Fig. 1h and Supplementary Fig. 1f).

C-terminal domain (CTD) of H1 linker subtypes is responsible of specific DNA binding affinity. H1B has the longest CTD as compared to the other isotypes, with 46 lysins among the 114 amino acids present in this domain [40]. This characteristic confers to H1B the stronger binding to the chromatin as compared to other H1 isotypes [6]. Moreover, H1B has been linked to the maintenance of MyoD-dependent myoblasts self-renewal, suggesting a potential similar effect in epidermal cells [9]. Thus, we focused mainly on the function of H1B.

### FOXM1-dependent expression of H1B

We previously identified the FOXM1 transcription factor as a determinant of holoclone-forming epidermal stem cells [21]. However, the molecular mechanism driving its function has not been further investigated. As the expression of H1B protein closely mirrors that of FOXM1 expression in holoclone- and meroclone-derived progeny (Fig. 1h) we explored whether FOXM1 could play a role in the expression of H1B. Co-expression levels of H1B and FOXM1 in the scRNA-seq dataset were analysed (Supplementary Fig. 2a). In cluster H, 68.2% of keratinocytes express both RNAs with a normalized expression of 0.42 for *FOXM1* and 3.76 for *H1B* (see ‘Methods’). In clusters M and P, they colocalize in only 18.3% and 6.5% of keratinocytes, respectively. Moreover, *FOXM1* normalized expression decreases to 0.25 (in cluster M) and 0.09 (in cluster P), *H1B* normalized expression decreases to 0.23 and 0.14 in clusters M and P, respectively.

To assess whether such interaction could be detected also in vivo (that is without a cultivation step), single-cell gene expression data were collected directly from an healthy donor-derived skin biopsy, immediately after cell extraction (see ‘Methods’, ref. [20] and Supplementary Fig. 2b). Upon samples integration, various subpopulations constituting the skin were identified, but only keratinocytes (defined based on *KRT14*, *KRT5*, *KRT10*, and *IVL* expression for a total of 7689 cells profiled) were retained for subsequent analysis. The Holoclone (H) cluster was clearly defined with high confidence (Supplementary Fig. 2c, d), based on *holoclone signature* expression levels (Supplementary Fig. 2e). Co-expression levels of *H1B* and *FOXM1* in the biopsy-derived dataset were analysed (Supplementary Fig. 2f). In cluster H, 38.7% of keratinocytes express both RNAs with a normalized expression of 0.46 for *FOXM1* and 2.22 for *H1B*. In clusters M and P, they colocalize in 4.4% and 0.2% of keratinocytes, respectively. Moreover, *FOXM1* normalized expression decreases to 0.13 and 0.01 in cluster M and P, respectively, while H1B normalized expression decreases to 0.21 and 0.02 in cluster M and P, respectively.

**Fig. 2:**
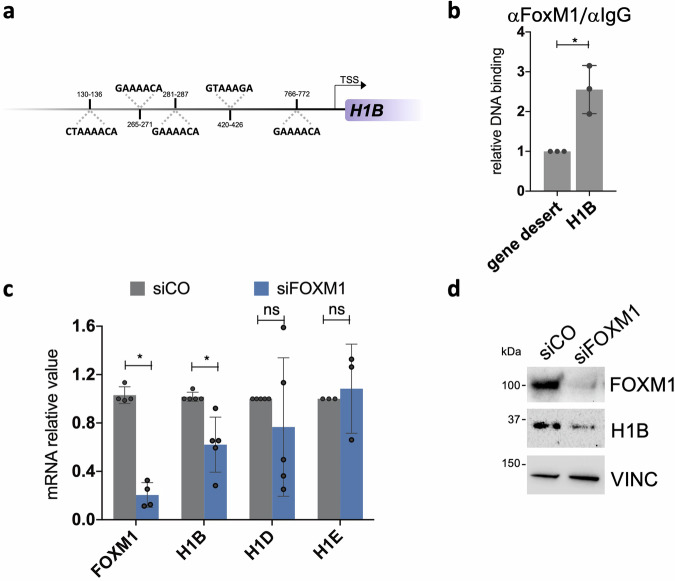
FOXM1 controls the expression of *H1B.* **a** Schematic representation of FOXM1 binding sites identified on *H1B* promoter. FOXM1 binding sites positions, and the corresponding sequences are shown. **b** ChIP-qPCR showing FOXM1 binding to the indicated sites in human primary keratinocytes. Relative DNA binding was calculated as a fraction of input and normalized with the negative region (gene desert, see Supplementary Table 4) arbitrarily set to 1, *N* = 3 independent experiments. *P < 0.05, Student t-test. **c** qRT-PCR quantification of the mRNA levels of *FOXM1* and *H1B, H1D* and *H1E* on cells derived from NHK cultures transfected with indicated siRNAs. Expression levels were normalized per *GAPDH* and given relative to the control (siCO) arbitrarily set to 1. Data are presented as mean +/− SD, *N* = at least 3 different independent biological replicates, *P < 0.05, Student t-test. **d** Western analysis on total cell extracts from cultures generated by NHK transfected with indicated siRNAs.

We investigated whether FOXM1 could bind to the promoter region of *H1B*, potentially driving its expression. Computational analysis showed five FOXM1 binding sites [41, 42] on *H1B* promoter (Fig. 2a and ‘Methods’). This notion was confirmed with chromatin immunoprecipitation experiments conducted on early passaged epidermal cultures derived from three healthy donors. Indeed, FOXM1 binds to the promoter region of *H1B*, suggesting *H1B* as a novel FOXM1-target gene (Fig. 2b). We then assessed the expression of *H1B* mRNA following a short-term depletion of FOXM1 through transient siRNA transfection. It is expected that the expression of a target would be affected even by a short-term (3 days) depletion of the inducer transcription factor. Consistent with this hypothesis, we observed reduced levels of *H1B* mRNA in siFOXM1-treated human primary keratinocytes, accompanied by a corresponding decrease in H1B protein expression (Fig. 2c, d), suggesting that *H1B* expression in human epidermal stem cells could be induced by FOXM1.

### H1B expression correlates with the presence of stem cells in human primary epidermal cultures

**Fig. 3:**
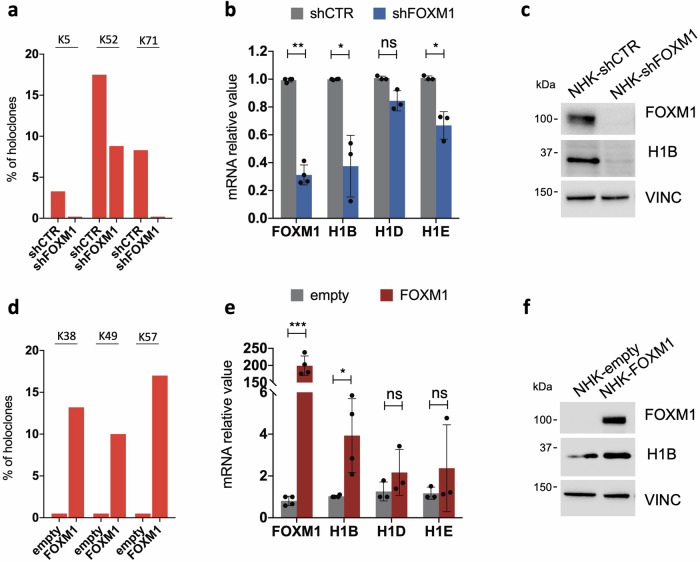
*H1B* expression correlates with the amount of epidermal stem cells. **a** Percentage of holoclones derived from 3 normal human keratinocytes cultures (NHK) [20] transduced with a lentiviral vector carrying a control shRNA (shCTR) or a shRNA targeting FOXM1 (shFOXM1). **b** qRT-PCR quantification of the mRNA levels of *FOXM1*, *H1B, H1D, H1E* on cells derived from NHK cultures transduced with a lentiviral vector carrying the indicated shRNA. Expression levels are normalized per *GAPDH*. Data are presented as mean +/− SD, *N* = at least 3 different independent biological replicates, *P < 0.05, **P < 0.01 Student t-test, ns = not significant. **c** Western analysis on total cell extract derived from NHK cultures transduced with lentiviral vectors carrying the indicated shRNAs. **d** Percentage of holoclones derived from 3 NHK cultures (see ‘Methods’) transduced with a lentiviral vector carrying an empty backbone as control (empty) or FOXM1 cDNA for FOXM1 overexpression (FOXM1). **e** qRT-PCR quantification of the mRNA levels of *FOXM1*, *H1B, H1D, H1E* on cells derived from FOXM1- (FOXM1) or empty backbone-transduced (empty) NHK cultures. Expression levels were normalized per *GAPDH*. Data are presented as mean +/− SD, *N* = at least 3 different independent biological replicates, *P < 0.05, ***P < 0.001 Student t-test, ns = not significant. **f** Western analysis on total cell extract derived from FOXM1- (FOXM1) or empty backbone-transduced (empty) NHK cultures. FOXM1 endogenous level in NHK-empty is not detectable due to technical reason. Molecular weight indicators are shown.

To gain further insights into the long-term interplay existing between FOXM1 and H1B, primary keratinocytes were stably transduced with a lentiviral vector expressing a FOXM1-targeting shRNA. Consistent with previous data [21], long-term downregulation of FOXM1 strongly reduced the percentage of holoclones in the culture (Fig. 3a). *H1B* emerged as the most downregulated H1 histone isoform in such cells, as compared to cells expressing the control shRNA (Fig. 3b). Accordingly, western blot analysis showed decreased levels of H1B in conditions where the stem cell compartment was significantly reduced by FOXM1 ablation (Fig. 3c). In contrast, FOXM1 enforced expression led to an increased proportion of stem cells (Fig. 3d, ref. [21]) along with elevated expression of H1 histone types, particularly the isoform *H1B* (Fig. 3e, f).

**Fig. 4:**
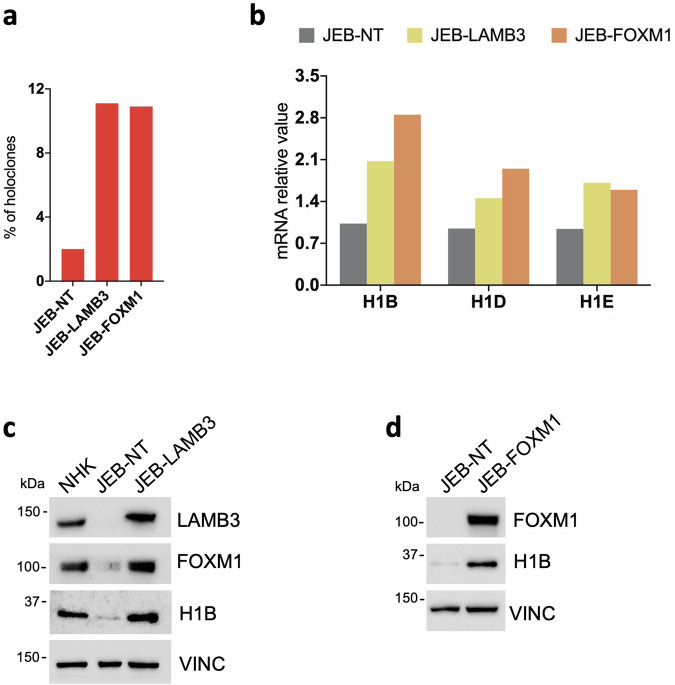
*H1B* expression in JEB-derived primary keratinocyte cultures. **a** Percentage of holoclones found in untransduced (JEB-NT), FOXM1- (JEB-FOXM1) or LAMB3-transduced (JEB-LAMB3) JEB-derived primary keratinocyte cultures (see ‘Methods’). **b** qRT-PCR quantification of the mRNA levels of *H1B, H1D, H1E* on cells derived from untransduced (JEB-NT), FOXM1- (JEB-FOXM1) or LAMB3-transduced (JEB-LAMB3) JEB-derived primary keratinocyte culture. Expression levels are normalized per *GAPDH*. **c** Western analysis on total cell extract derived from NHK, untransduced (JEB-NT) and LAMB3-transduced (JEB-LAMB3) JEB-derived primary keratinocyte cultures. Molecular weight indicators are shown. **d** Western analysis on total cell extract derived from untransduced (JEB-NT), FOXM1- (JEB-FOXM1) JEB-derived primary keratinocyte cultures. Molecular weight indicators are shown.

Epidermal stem cells are firmly attached to the underlying basement membrane through the interaction of ɑ6β4 integrins with the basal lamina component laminin 332, a heterotrimeric protein composed of α3, β3 and γ2 chains, which are encoded by *LAMA3*, *LAMB3* and *LAMC2*, respectively [43–45]. The binding of ɑ6β4 integrins to laminin 332 induces nuclear localization of YAP, a co-transcription factor critical for the self-renewal of epidermal stem cells [22]. Such interaction is dismantled in patients affected by JEB, a devastating, often early lethal, genetic skin diseases marked by extensive skin blistering [46, 47]. Hence, primary keratinocyte culture derived from patients suffering from *LAMB3*-dependent JEB are marked by an accelerated stem cell exhaustion due to inactivation of YAP and its downstream targets, including FOXM1 [21, 22]. To strengthen the notion that high levels of Histone H1 linker subtypes correspond to stem cell-enriched conditions, we modulated the stem cell content in cells obtained from a JEB new born, a double-heterozygous carrier of two mutations (c.2242 G > T and c.823-1 G > T) in the LAMB3 gene. Of note, both *LAMB3* gene therapy and enforced *FOXM1* in *LAMB3*-dependent JEB-derived human primary keratinocytes restored the proper amount of stem cells (Fig. 4a) and the expression of H1 linker subtypes, particularly *H1B* (Fig. 4b–d).

### H1B silences the expression of differentiation markers

**Fig. 5:**
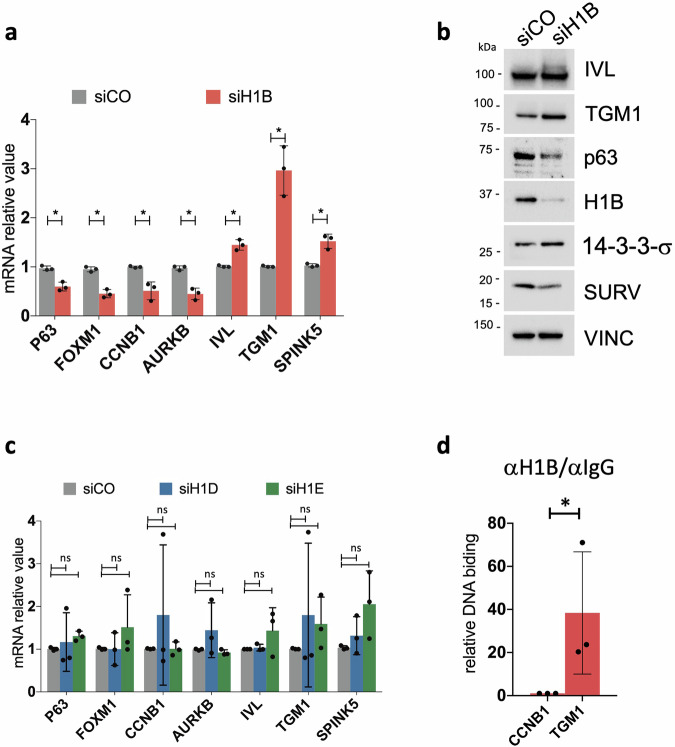
H1B silences the expression of differentiation genes. **a** qRT-PCR quantification of the mRNA levels of some genes comprised in the *Holoclone signature* (*p63, FOXM1, CCNB1, AURKB*) and of a differentiation-related gene (*TGM1, IVL* and *SPINK5*) in NHK transfected with a control siRNA (siCO) or a siRNA specific for *H1B* (siH1B). Expression levels were normalized per *GAPDH*. Data are presented as mean +/− SD, *N* = 3 different independent biological replicates, *P < 0.05, Student t-test. **b** Western analysis on total cell extracts derived from NHK cultures transfected with indicated siRNAs. Molecular weight indicators are shown. **c** Same of (**a**) for *H1D* and *H1E*. **d** ChIP-qPCR showing H1B binding to the indicated sites in human primary keratinocytes. DNA binding value relative to IgG was calculated as a fraction of input of the corresponding gene and normalized with CCNB1 promotorial region arbitrarily set to 1; Data are presented as mean +/− SD, *N* = 5 different independent biological replicates. *P < 0.05, Student t-test.

To directly assess the effect of H1B deletion, we targeted H1 isoforms using specific siRNAs. Given the high conservation in the genomic sequence of the coding sequence for H1 linker isoforms, we initially confirmed that only *H1B*, *H1D*, and *H1E* expression was specifically affected in cells treated with their corresponding siRNAs (Supplementary Fig. 3). Of note, only the downregulation of *H1B* led to a reduced expression of genes associated with the holoclone signature (*p63, FOXM1, CCNB1, AURKB*), coupled with increased expression of differentiation-related genes such as *TGM1, IVL, SPINK5* and 14-3-3-σ (Fig. 5a, b). Similar effects were not observed upon depletion of *H1D* or *H1E* (Fig. 5c), confirming that H1B plays a specific role in modulating self-renewal and differentiation markers.

H1 histone linker plays a crucial role in DNA compaction wrapped around core histones, thereby leading to the silencing or repression of genes within those genomic regions [2, 4, 40]. To determine where H1B exploits its function in the keratinocyte genome, we took advantage of chromatin immunoprecipitation experiments. In conditions enriched with stem cells, where *H1B* is highly expressed, we observed that it is weakly associated with promoter of genes included in the holoclone signature (such as *CCNB1*). Conversely, H1B is strongly associated with the promoter of genes linked to keratinocyte differentiation (such as *TGM1*) (Fig. 5d).

**Fig. 6:**
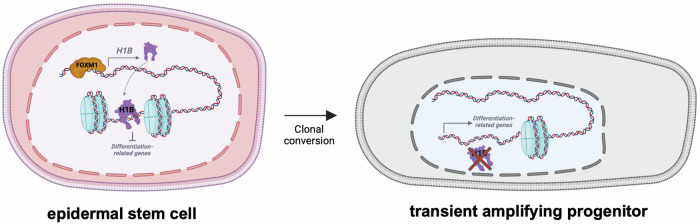
Schematic representation of the proposed mechanism. In epidermal stem cells, FOXM1 transcriptionally activates *H1B*. H1B binds to promotorial regions of differentiation-related genes, inhibiting their transcription. During clonal conversion epidermal stem cell gives rise to transient amplifying cells where the reduced expression of H1B leads to the derepression of differentiation-related genes. The figure was created with BioRender.com.

Altogether, these findings suggest that H1B plays an important role in sustaining FOXM1-dependent self-renewal of human epidermal stem cells. Acting as an epigenetic modifier, H1B binds the promotorial region of genes involved in the differentiation process to maintain their silencing (Fig. 6).

## Discussion

The skin is one of the organs of the human body that is constantly renewed throughout the entire life [48]. Therefore, the biochemical networks that control the balance between self-renewal and differentiation in human primary keratinocytes must be finely tuned.

Autologous epidermal cultures have been used for the last 30 years in regenerative medicine to treat burned patients or patients affected by severe genetic skin diseases [12–16, 18, 49–51]. The successful outcome of such clinical approaches relies on the presence of an adequate number of epidermal stem cells in the graft, able to guarantee the long-lasting regeneration of the epidermis [16, 52]. Gaining more knowledge in the networks controlling self-renewal would allow a better control of epidermal stem cell content in cultured epidermal grafts.

Histone H1 is mostly known as a structural protein responsible for chromatin compaction, however, it plays a pivotal role also in regulating differentiation and gene transcription [2, 53]. Interestingly, it has been proven that the overall H1 levels progressively increase during the differentiation of ES cells [54, 55], while we observed the opposite during the process of clonal conversion and differentiation of the epidermis. Indeed, we found that four H1 isoforms (*H1B*, *H1C*, *H1D* and *H1E*) are more expressed in epidermal stem cells as compared to transient amplifying progenitors, hence their expression decreases during epidermis differentiation.

We have shown that H1B acts downstream of FOXM1 in normal human keratinocytes. Short- and long-term FOXM1 modulation results in a coherent modulation of *H1B* expression, mirroring the amount of stem cells in the culture. We found that in epidermal stem cells H1B binds to genes involved in differentiations, silencing their expression. Conversely, it is not found associated with genes required for self-renewal that are actively transcribed. Remarkably, we observed that when H1B is downregulated, there is a decrease in the expression of its activator, *FOXM1*. Consequently, the transcription of *H1B* is even more reduced, preventing the silencing of genes associated with differentiation. As a result, the differentiation process is further sustained. These findings shed light on one possible downstream mechanism that maintains self-renewal along the YAP-FOXM1 axis previously identified [21, 22].

The importance of histone H1 in regulating gene expression is suggested also by the existence of many different isoforms that have specific roles depending on the cell type, tissue and differentiation stage [7, 9–11, 56]. Even though H1B, H1D and H1E have high sequence similarity suggesting a possible functional redundancy, we observed that only loss of H1B causes a concomitant effect in decreasing self-renewal-related genes and increasing differentiation genes. Thus, we unveil a new specific function of H1B, like the one already observed during skeletal muscle development [9].

These findings, together with recent insights on the importance of epigenetic regulation in epidermis [57], allow a better understanding of the fine interplay between self-renewal and differentiation in epidermal stem cells, driving a better development of stem-cells based ex vivo cells and gene therapy applications for different types of genetic skin diseases.

### Supplementary information

Supplementary File

Supplementary Figure 1

Supplementary Figure 2

Supplementary Figure 3

original data file, uncropped western blots

## Data Availability

scRNA-seq data refers to article Enzo et al. [21] have been deposited in the Gene Expression Omnibus database under accession code: GSE155817. We declare that the data supporting the findings of this study are available within the paper and from the authors upon request.
