# Supplementary material for: Biochemical role of FOXM1-dependent histone linker H1B in human epidermal stem cells
Source: Cell Death Dis. 2024 Jul 17;15(7):508. doi: 10.1038/s41419-024-06905-1 (PMC11255229; doi:10.1038/s41419-024-06905-1)
Supplement: Supplementary file 1 — Supplementary File [file 41419_2024_6905_MOESM1_ESM.docx]

**Biochemical role of FOXM1-Dependent Histone**

**Linker H1B in human Epidermal Stem Cells**

Maria Pia Polito^1^, Grazia Marini^1^, Alessandra Fabrizi^1^, Laura Sercia^1^, Elena Enzo^1*^ and Michele De Luca^1*^

^1^ Centre for Regenerative Medicine “Stefano Ferrari”, Department of Life Science, University of Modena and Reggio Emilia, Modena, Italy

*These Authors equally contributed to this work

Co-Corresponding authors: [elena.enzo@unimore.it](mailto:elena.enzo@unimore.it) and michele.deluca@unimore.it

**Supplementary files**

The article “Biochemical role of FOXM1-Dependent Histone Linker H1B in human Epidermal Stem Cells” by Polito et al, contains the following Supplementary files:

- Supplementary Figure legends (pag. 2)
- Supplementary Materials (pag. 3)
- Supplementary Tables (pag. 3)

**Supplementary figures legends:**

**Supplementary Fig. 1 Expression of other histone H1 linker subtypes in single cell dataset. (a)**Violin plots showing the expression of *HIST1H1A*, *H1F0* and *H1FX* among the 5 clusters identified in scRNA-seq. **(b)** Serial cultivation of normal human keratinocytes (NHK). Percentage of clonogenic cells (dark red line) was calculated as the ratio between grown colonies and plated cells. Percentage of aborted colonies (grey line) was calculated as the ratio between the colonies scored as aborted and the number of clonogenic cells. (**c)** Same as (b). **(d)** qRT-PCR quantification of the mRNA levels of five different H1 isoforms (*H1A, H1B, H1C, H1D, H1E*) and two differentiation markers (*IVL* and *SPINK5*) on NHK collected during serial cultivation. Expression levels were normalized per *GAPDH*. **(e)** Same as (d). **(e)** Western analysis of total cell extracts from cultures generated by holoclones (H1 and H2), meroclones (M1 and M2), and paraclone (P1) isolated by clonal analysis (see “Methods”) of sub-confluent NHK. Molecular weight indicators are shown.

**Supplementary Fig. 2 Colocalization of H1B and FOXM1 in scRNA datasets. (a)** Feature plot showing co-expression of FOXM1 and HIST1H1B in the dataset from *in vitro* cultured keratinocytes. **(b)** UMAP of the single-cell RNA-seq dataset obtained from a healthy-donor-derived skin biopsy. Only keratinocytes are shown in the graph. **(c)** DotPlot showing expression of clonogenic, holoclone, and differentiation markers used to annotate the five keratinocyte clusters identified. **(d)** Violin plots depicting clusters prediction confidence and (**e**) holoclone signature expression levels in keratinocytes clusters identified within skin biopsy dataset. In boxplots, lines in the middle of boxes correspond to median values. Lower and upper hinges correspond to the first and third quartiles, the upper whisker extends from the hinge to the largest value no further than 1.5 × IQR (inter-quartile range) from the hinge. The lower whisker extends from the hinge to the smallest value at most 1.5 × IQR of the hinge.

**(f)** Feature plot showing co-expression of FOXM1 and HIST1H1B in the biopsy dataset.

**Supplementary Fig. 3 Expression of histone H1 linker mRNAs after isoform specific silencing.** qRT-PCR quantification of the mRNA levels of *H1A*, *H1B*, *H1C*, *H1D* and *H1E* in NHK transfected with a control siRNA (siCO) or a siRNA specific for *H1B, H1D* and *H1E*. Expression levels were normalized per *GAPDH*. Average and standard deviation are displayed. Data are presented as mean +/- SD, N = at least 2 different independent biological replicates.

**Supplementary Materials**

Uncropped blots and densitometric analysis of Western Blots. The density of the Region of Interest was measured with Image Lab Software version 6.1.0 by Bio-Rad srl. Values are given as the ratio between the given marker and the endogenous controls (VINC or GAPDH).

**Supplementary Tables:**

**Supplementary table 1 - List of antibodies used, source and, concentration**

| Antibody | Company | Catalog Number | Description | Western Blot | ChIP |
| --- | --- | --- | --- | --- | --- |
| anti-FOXM1 | Cell signaling Technology | D3F2B | Rabbit monoclonal | 1/1000 | 1/30 |
| anti-p63 alfa | Di Iorio et al., 2005 | N/A | Rabbit monoclonal | 1/2000 |  |
| anti-VINCULIN | Sigma-Aldrich | V4505 | Mouse monoclonal | 1/10000 |  |
| anti-HIST1H1B | Abcam | ab18208 | Rabbit polyclonal | 1/50000 | 1/300 |
| Anti-LAMB3 | Santa Cruz Biotechnology | sc-7651 | Goat polyclonal | 1/700 |  |
| anti-TGM1 | Invitrogen | PA5-59088 | Rabbit polyclonal | 1/4000 |  |
| anti-IgG | Abcam | ab171870 | Rabbit monoclonal |  | 1/300 |
| Anti-IVL | Thermo Fischer Scientific | Ma1-25752 | Mouse Monoclonal | 1:8000 |  |
| Anti-14-3-3-σ | Abcam | Ab14123 | Mouse Monoclonal | 1/200 |  |
| Anti ITGB4 | Santa Cruz Biotechnology | Sc-135950 | Mouse Monoclonal | 1/200 |  |
| Donkey anti-mouse IgG HRP | Abcam | ab98665 |  | 1/200000 |  |
| Goat anti-rabbit IgG HRP | Abcam | ab205717 |  | 1/2000 - 1/5000 |  |

**Supplementary Table 2 - List of siRNA used with code, type and, catalogue number.**

| siRNA name | Gene | Code | Type | Catalog number |
| --- | --- | --- | --- | --- |
| siCO | negative control | - | Silencer select (Thermo Fisher) | 4390843 |
| siFOXM1 | *FOXM1* | s5250 | Silencer select (Thermo Fisher) | 4392420 |
| siH1B | *HIST1H1B* | s6404 | Silencer select (Thermo Fisher) | 4427037 |
| siH1D | *HIST1H1D* | s6399 | Silencer select (Thermo Fisher) | 4427037 |
| siH1E | *HIST1H1E* | s6401 | Silencer select (Thermo Fisher) | 4427037 |

**Supplementary Table 3 – List of Taqman probes used with catalogue numbers.**

| Gene | Catalogue Number |
| --- | --- |
| *TP63* | HS00978340_m1 |
| *CCNB1* | Hs01030099_m1 |
| *FOXM1* | Hs01073586_m1 |
| *GAPDH* | 4352665 |
| *AURKB* | HS00945855_g1 |
| *HIST1H1A* | HS00271225_s1 |
| *HIST1H1B* | HS00271207_s1 |
| *HIST1H1C* | HS00271185_s1 |
| *HIST1H1D* | HS00271187_s1 |
| *HIST1H1E* | HS00271195_s1 |
| *IVL* | HS00846307_s1 |
| *TGM1* | HS00165929_m1 |
| *SPINK5* | HS00928570_m1 |

**Supplementary Table 4 - List of primers used with specified name, sequence, reference, and application.**

| Primer name | Sequence | Reference | Application |
| --- | --- | --- | --- |
| TGM1-FOR | CACAGCTGGCCCACACCCCA | custom made | ChIP |
| TGM1-REV | GAGTCCAGGCGGCCCACACT | custom made | ChIP |
| H1B-FOR | GTTGCCTTCTTCTTAGCCGG | custom made | ChIP |
| H1B-REV | AGTTTCTTGCCACCATGTCG | custom made | ChIP |
| CCNB1-FOR | CGCGATCGCCCTGGAAACGCA | custom made | ChIP |
| CCNB1-REV | CCCAGCAGAAACCAACAGCCGT | custom made | ChIP |
| Gene_desert_FOR | AACGCTCAAATCTCCCACAG | custom made | ChIP |
| Gene_desert_REV | AAAGAAGGGACGAAG | custom made | ChIP |
