# Supplementary figures and images for: Biochemical role of FOXM1-dependent histone linker H1B in human epidermal stem cells
Source: Cell Death Dis. 2024 Jul 17;15(7):508. doi: 10.1038/s41419-024-06905-1 (PMC11255229; doi:10.1038/s41419-024-06905-1)

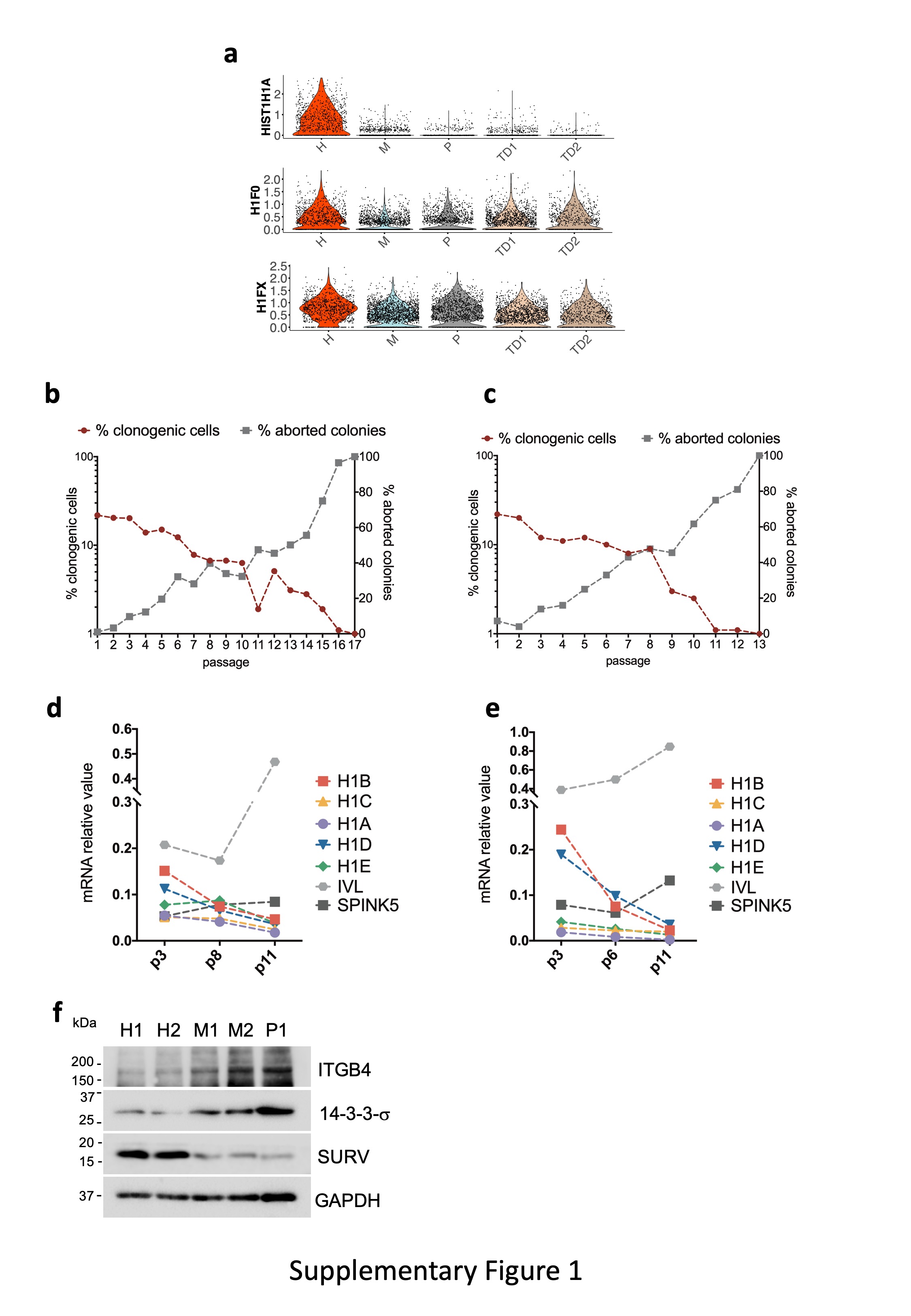

Supplement: Supplementary file 2 — Supplementary Figure 1 [file 41419_2024_6905_MOESM2_ESM.jpg]

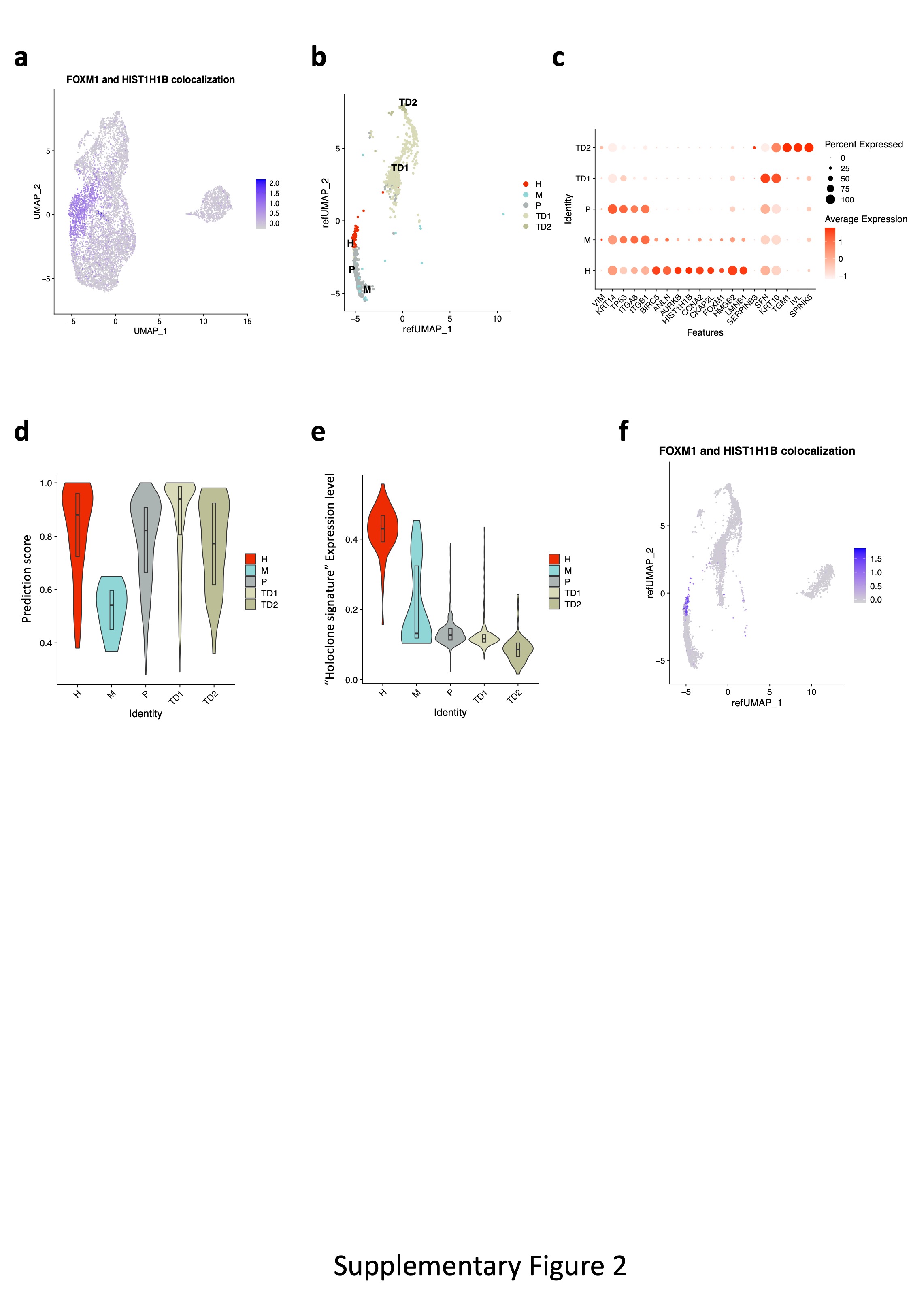

Supplement: Supplementary file 3 — Supplementary Figure 2 [file 41419_2024_6905_MOESM3_ESM.jpg]

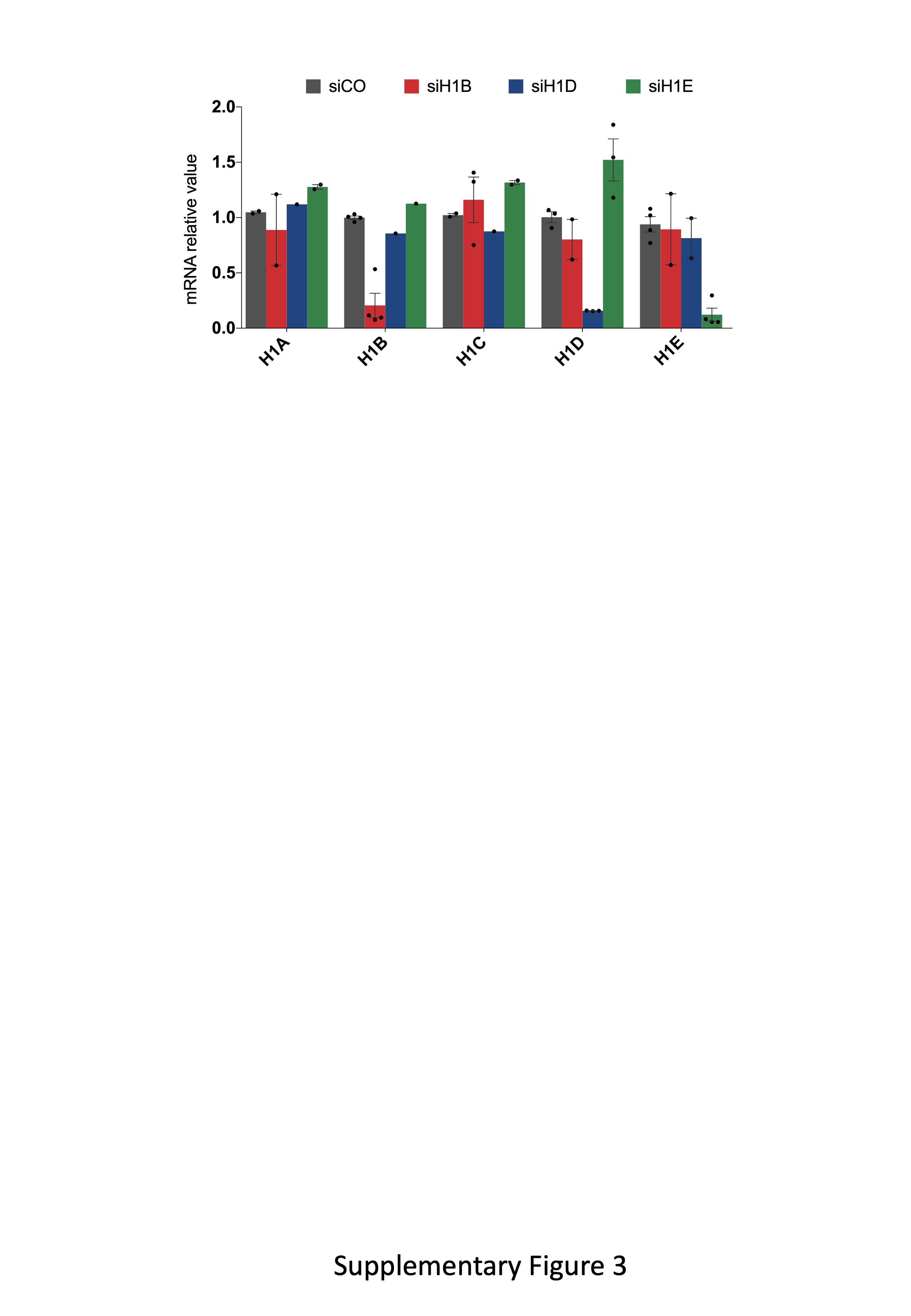

Supplement: Supplementary file 4 — Supplementary Figure 3 [file 41419_2024_6905_MOESM4_ESM.jpg]
