# Supplementary material for: Biochemical role of FOXM1-dependent histone linker H1B in human epidermal stem cells
Source: Cell Death Dis. 2024 Jul 17;15(7):508. doi: 10.1038/s41419-024-06905-1 (PMC11255229; doi:10.1038/s41419-024-06905-1)

Figure 1h

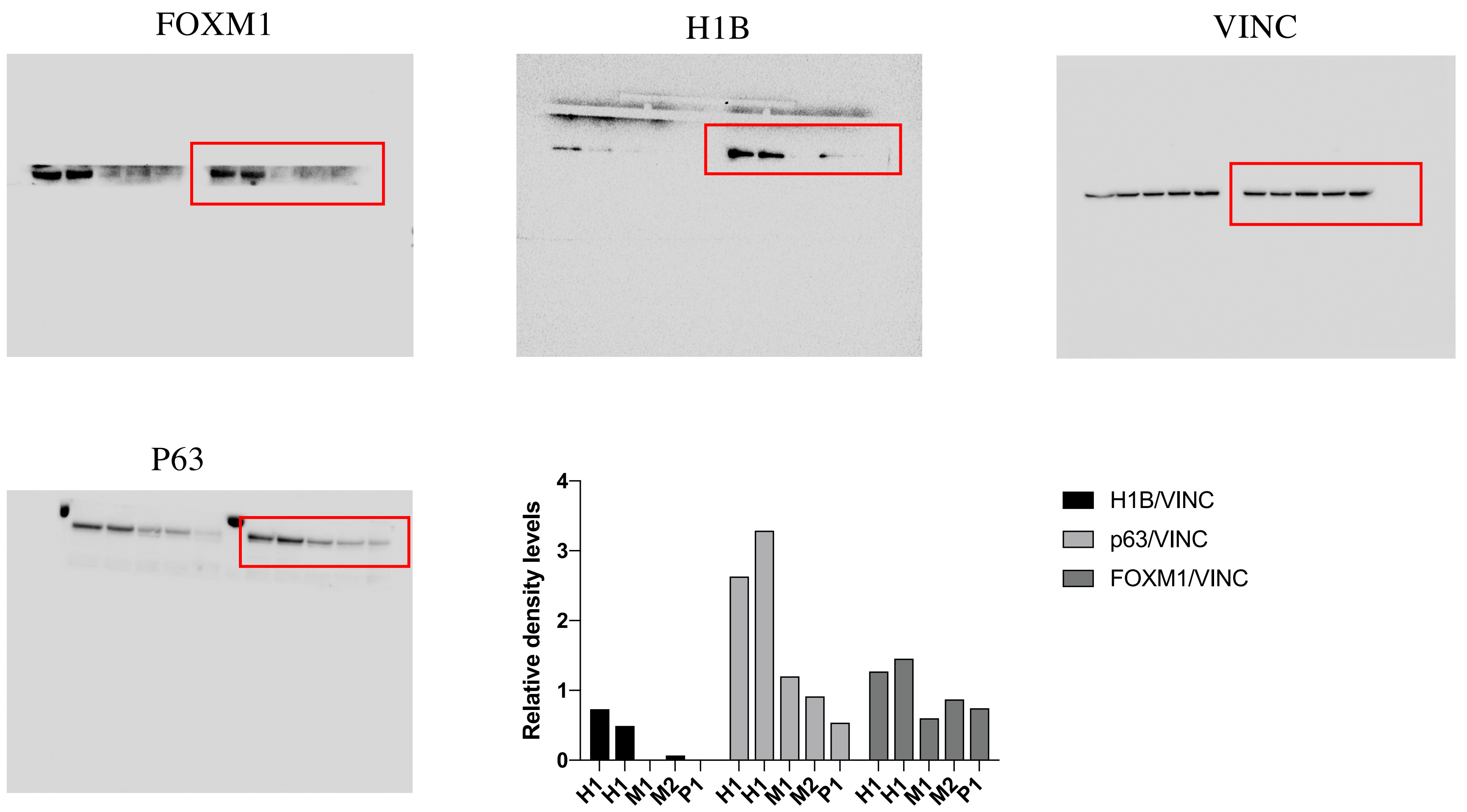

FOXM1
H1B
VINC
P63
Relative density levels
0
1
2
3
4
H1 H1 M1 M2 P1 H1 H1 M1 M2 P1 H1 H1 M1 M2 P1
H1B/VINC
p63/VINC
FOXM1/VINC

Figure 2d

FOXM1

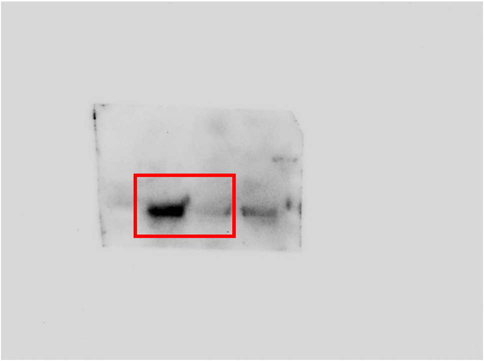

H1B

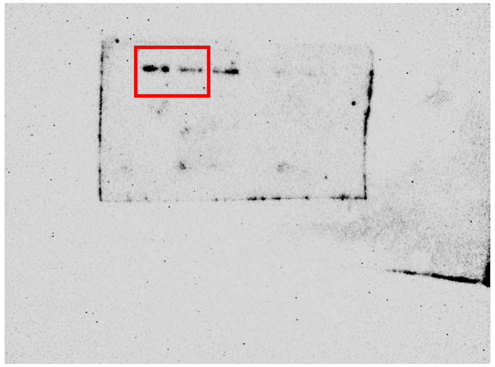

VINC

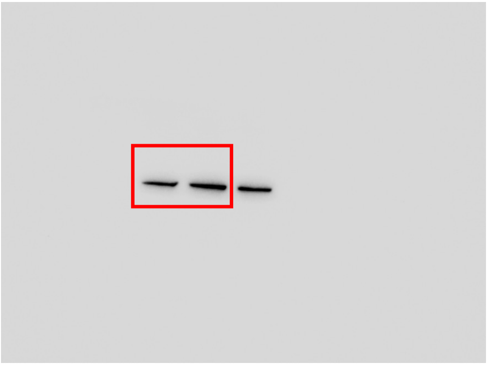

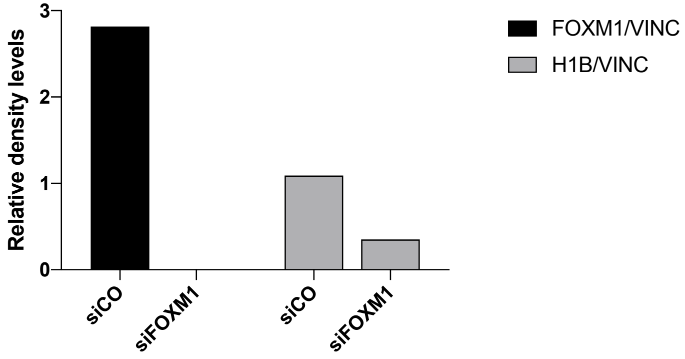

Figure 3c

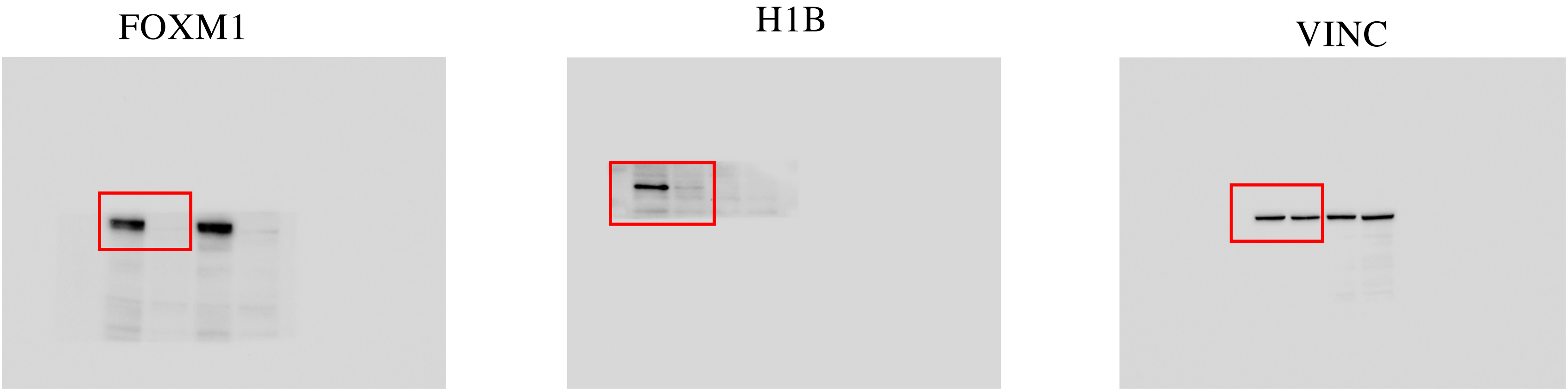

FOXM1
H1B
VINC

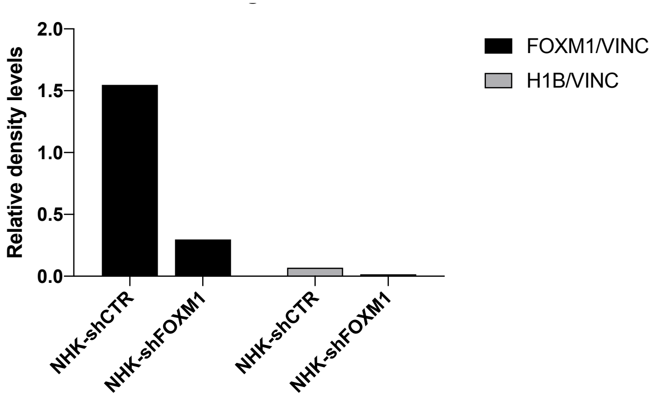

Relative density levels
2.0
1.5
1.0
0.5
0.0
NHK-shCTR
NHK-shFOXM1
NHK-shCTR
NHK-shFOXM1
FOXM1/VINC
H1B/VINC

Figure 3f

FOXM1

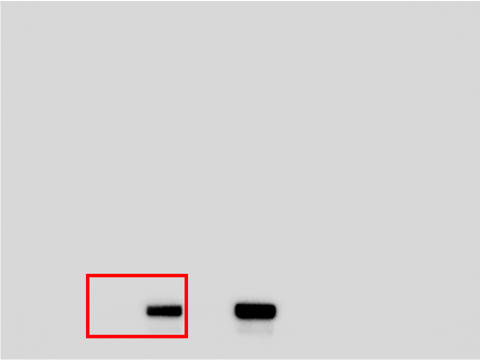

H1B

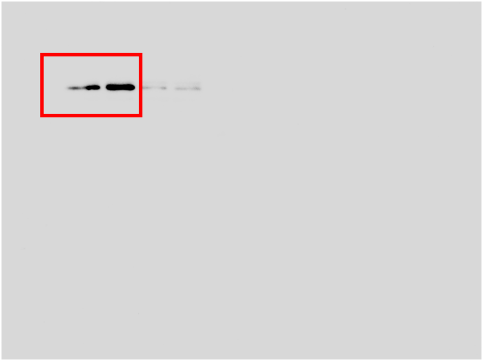

VINC

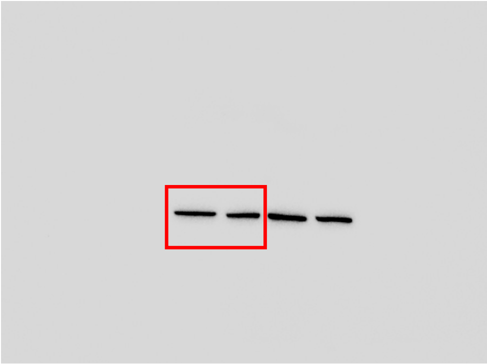

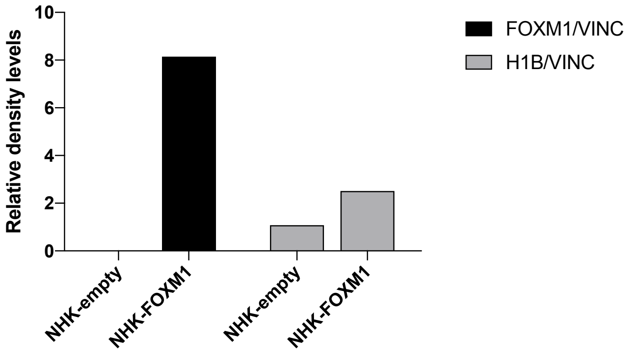

Figure 4c

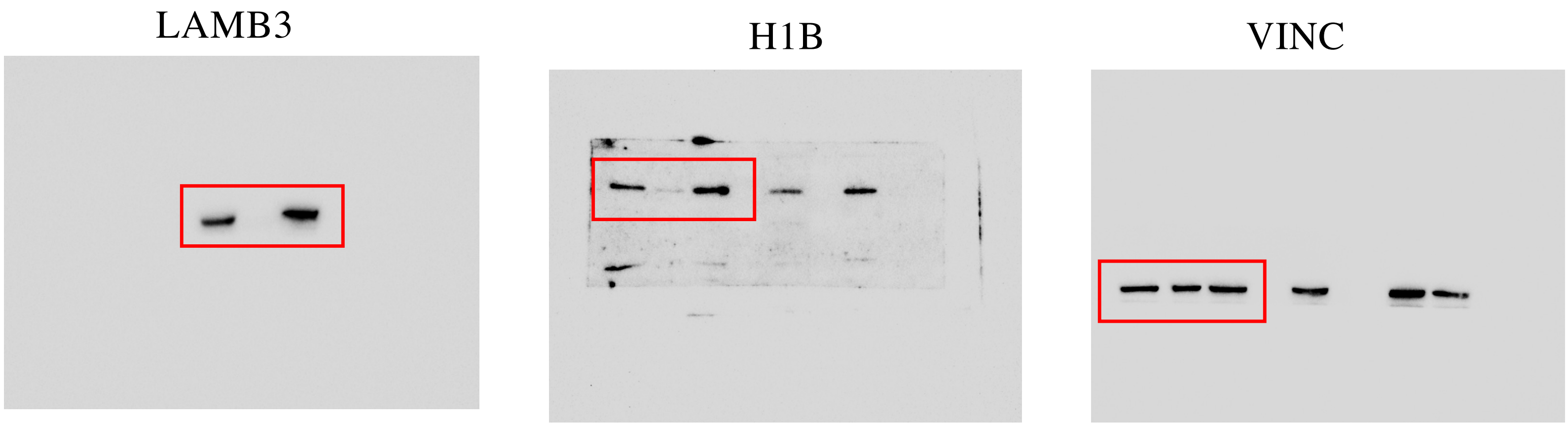
LAMB3
H1B
VINC

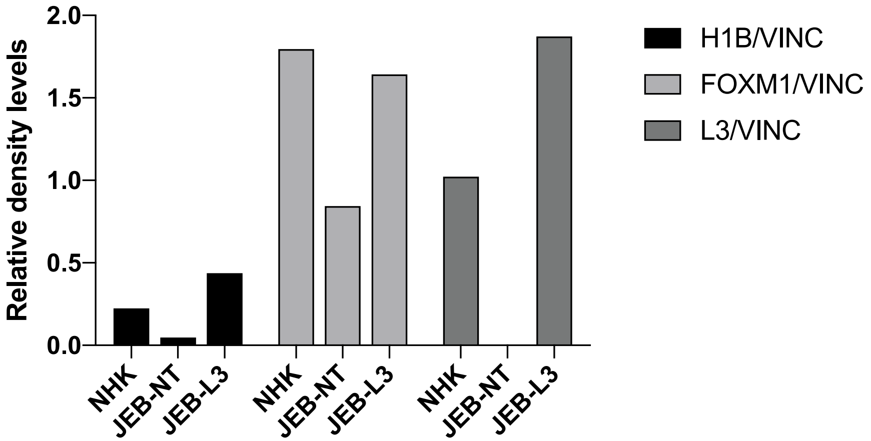
Relative density levels
2.0
1.5
1.0
0.5
0.0
NHK
JEB-NT
JEB-L3
NHK
JEB-NT
JEB-L3
NHK
JEB-NT
JEB-L3
H1B/VINC
FOXM1/VINC
L3/VINC

Figure 4d

FOXM1

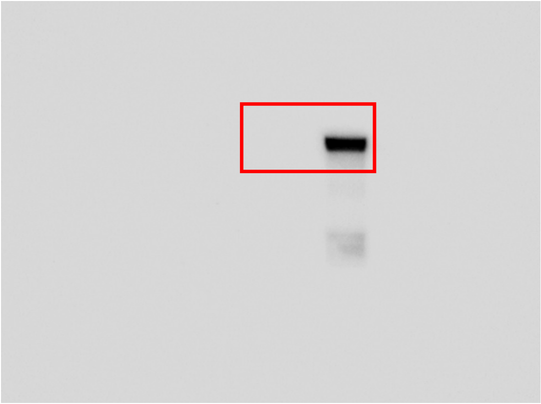

H1B

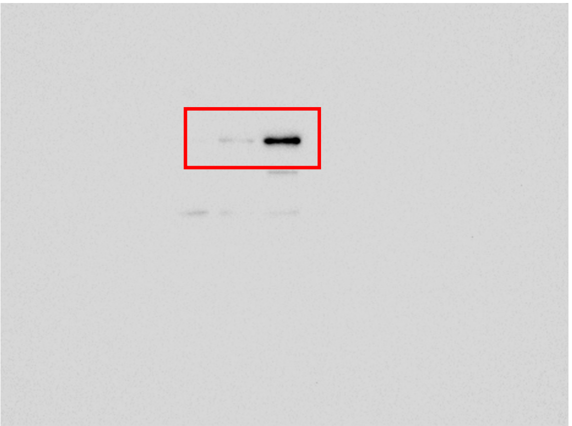

VINC

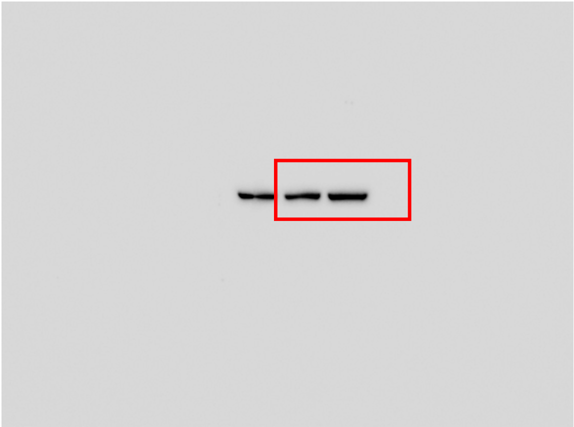

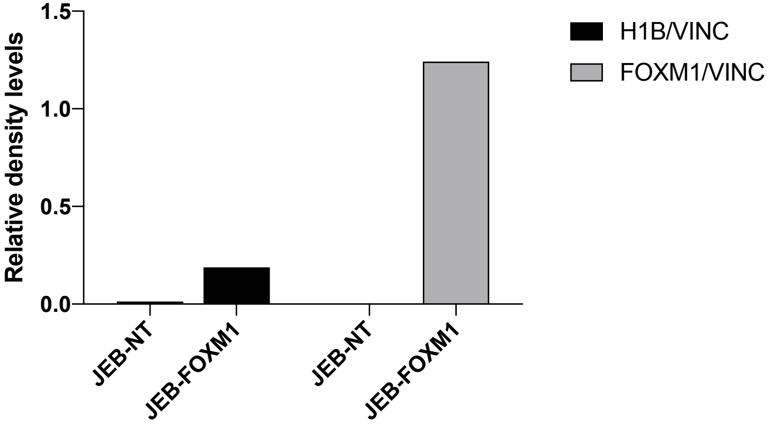

Figure 5b

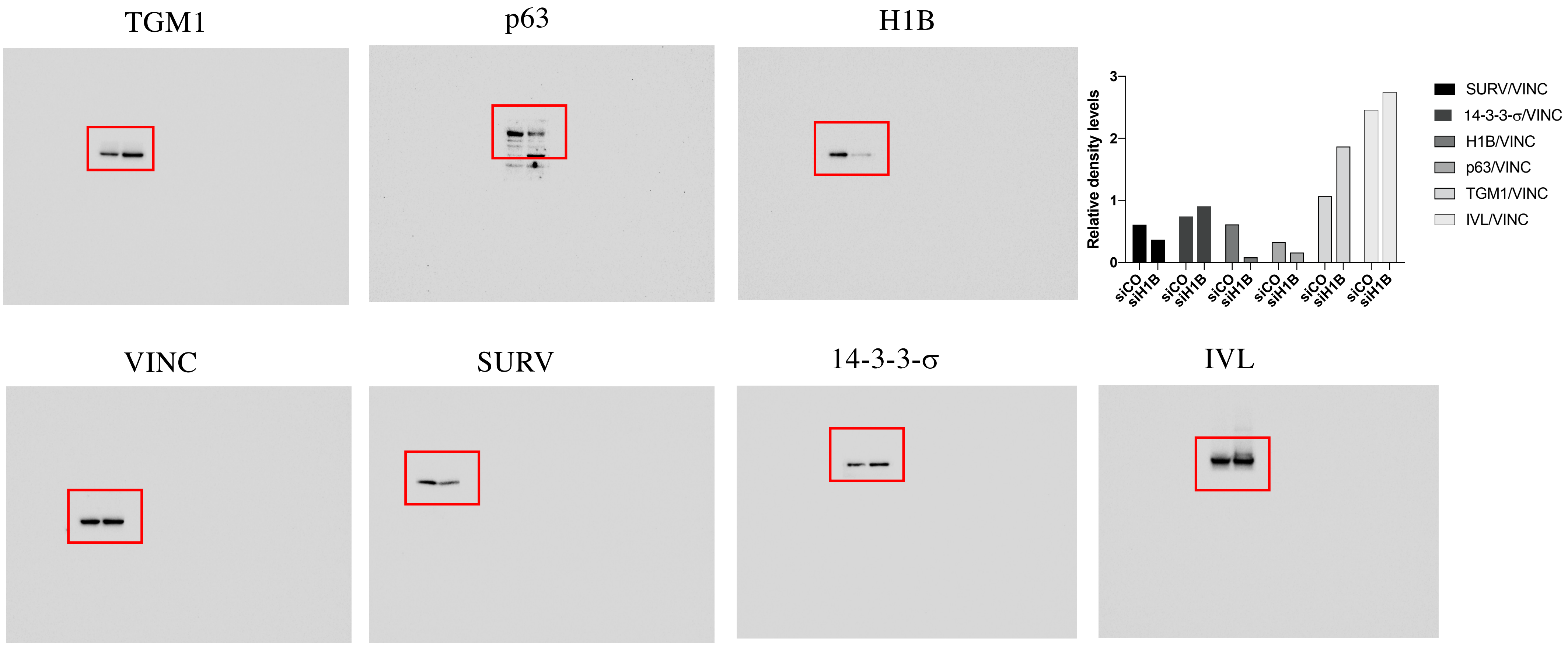
TGM1
p63
H1B
Relative density levels
3
2
1
0
siCO
siH1B
SURV/VINC
14-3-3-σ/VINC
H1B/VINC
p63/VINC
TGM1/VINC
IVL/VINC
VINC
SURV
14-3-3-σ
IVL

Supplementary Fig. 1f

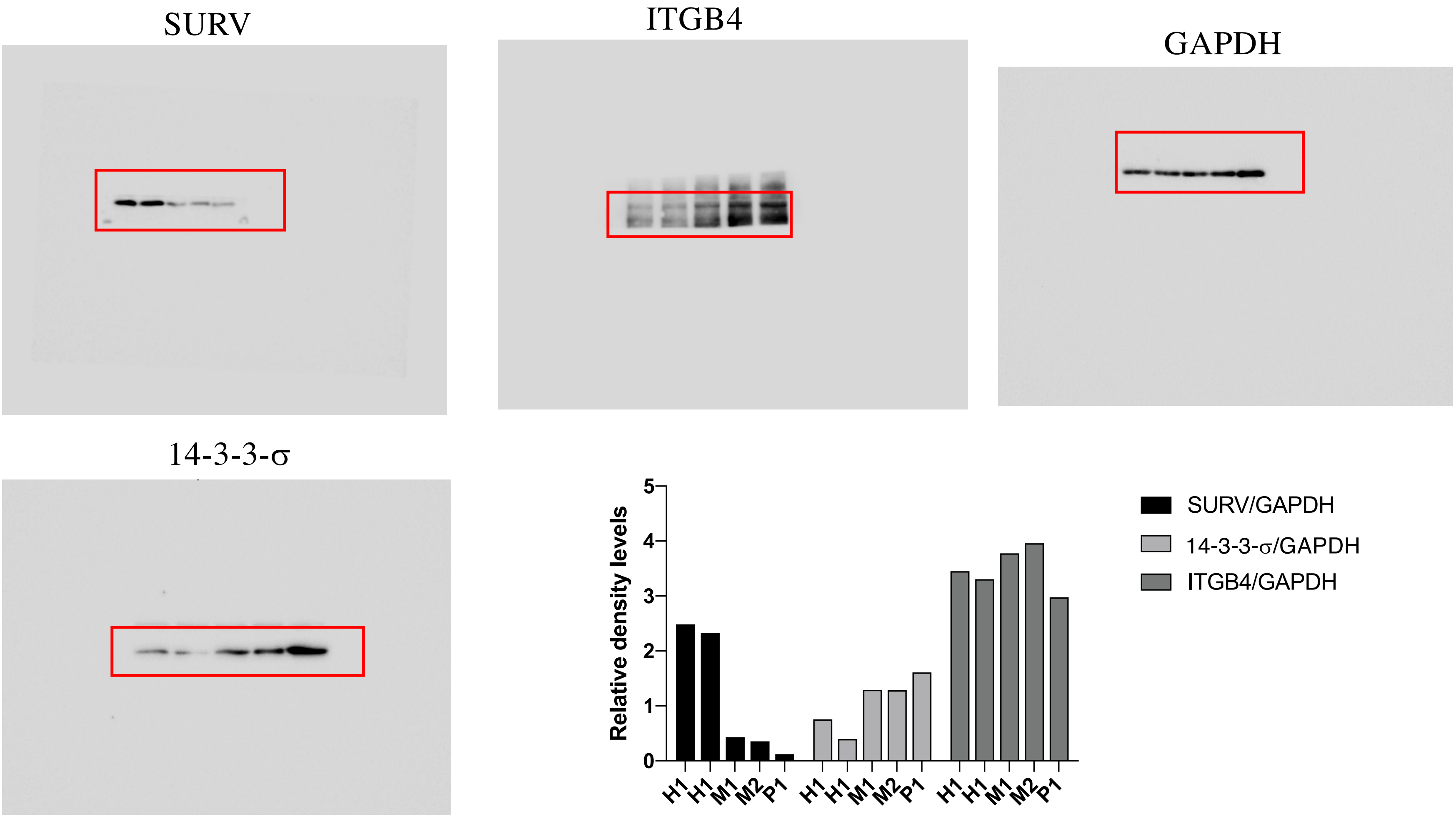

Supplement: Supplementary file 5 — original data file, uncropped western blots [file 41419_2024_6905_MOESM5_ESM.pdf]
